# Macromorphological findings in cases of death in water: a critical view on “drowning signs”

**DOI:** 10.1007/s00414-020-02469-9

**Published:** 2020-11-25

**Authors:** Simon Schneppe, Martin Dokter, Britta Bockholdt

**Affiliations:** Institute of Legal Medicine, University Medicine of Greifswald, Kuhstraße 30, 17489 Greifswald, Germany

**Keywords:** Drowning, Putrefaction, Emphysema aquosum, Svechnikov sign, Foam, Autopsy diagnoses

## Abstract

Death in water is a challenging issue in forensic pathology since from natural death to homicide all circumstances of death in water are conceivable. Therefore, the correct interpretation of all abnormal autopsy findings is important. In order to determine a death by drowning, numerous internal and external signs of drowning are already described. However, these are supposed to be influenced by various factors reducing their significance and evidence. Moreover, the autopsy of water corpses often reveals further pathological findings that should not be underestimated for determining the cause of death. The aim of this study was to set frequencies of the observed drowning signs in context to the forensic literature and to identify possible influencing factors. In this study, we observed that pathological organ changes of the cardiovascular system were significantly more common in corpses after shortened (atypical) drowning processes than in classical drowned victims. Furthermore only a complete formation of external foam, immediately after the corpse’s recovery, was exclusively found in drowning victims. All other drowning signs were either also observed in non-drowning deaths in water or no information could be provided with reasonable assurance. In addition, many of the examined drowning signs were negatively affected by prolonged postmortem intervals, putrefaction, or resuscitation attempts. It can be concluded from our analysis that morbidity is an important factor in deaths in water. Morbidity can support a death by drowning in case of incidents in water. For the examined drowning signs, no high diagnostic certainty could be observed. Nevertheless, these findings can increase their diagnostic value—if forensic physicians take influencing factors into consideration.

## Introduction

In forensic pathology, death in water, a very multifaceted issue, represents a challenge for the forensic physician. Since all circumstances, from natural death to homicide, are conceivable in water, critical remarks and logical classification of all abnormal findings made in autopsy are of great importance [1, 2].

Drowning is considered to be the leading cause of death in water and the third most common cause of accidental death worldwide, with the highest drowning rates in developing countries [3, 4]. The World Health Organization (WHO) defines it as follows: “The process of experiencing respiratory impairment from submersion/immersion in liquid” [5]. A precondition for this respiratory disability is that the airway openings must be on a level with the air-liquid boundary layer [6, 7].

The process of drowning can be divided into five stages: *reflective inspiration*, *willful apnea*, *dyspnea*, *convulsion*, and *terminal apnea* [7, 8]. During the drowning process, the stages cause characteristic changes to the organ systems, which can be identified as signs of drowning in the autopsy. “Atypical drowning” must be distinguished from “classical drowning.” Atypical drowning is defined as a drowning process that has been shortened by water-independent factors might cause partial less expressed drowning signs. These include, for example, nerval reflexes and mechanical effects of force [7, 9]. This definition is inconsistent throughout the world.

During the stage of dyspnea, the increased respiratory stimulus leads to uncontrolled fluid inflow into the airways and sinuses. The detection of fluid in the sphenoid sinus was first described in 1965 by V.A. Svénikov and is since used as “Svechnikov sign” for the diagnosis of death by drowning [10]. Therefore, the upper side of the sphenoid sinus is punctured with a needle and the contents can finally be aspirated [11, 12]. Also on the skull, Niles (1963) described hemorrhaging into the middle ear cavities and blood-stained mastoid processes, which are likely due to pressure changes during the drowning process [10, 13].

An important vital drowning sign is external foam, resulting from the mixture of air, water, bronchial mucosa secretions, and surfactant in the lungs [8, 9, 14]. The foam passes out the airways retrogradely and can be observed as a typical fungiform structure in front of the mouth and nostrils [15].

Inside the stomachs of drowning victims, Fritz (1932) found longitudinal mucosa lesions, attributed to the forced vomiting of large amounts of liquid and foam [9, 16–18]. In addition, Wydler (1896) described a three-layering of a foamy upper phase which consists of a mixture of drowning fluid and tracheal secretion, lying on a medium liquid phase and food particles at the bottom [10, 19].

Signs of excessive extension at the stage of convulsion may be hemorrhages into the respiratory muscles of the neck [9, 20].

The most important sign of freshwater drowning in the lung is the occurrence of a dry pulmonary emphysema, the so-called emphysema aquosum [14, 21]. Increased phlegm production and foam formation create a valve mechanism by expiratorily closing the airways, resulting in a hyperinflation of the lungs. These mechanisms create the image of ballooned lungs filling the pleural cavities and reaching over the pericardium with their edges [14, 22]. Imprints of the ribs on the lung surface can often be seen [8, 9, 23].

Histologically, the overinflated lungs present flattened and ruptured interalveolar septa, surrounding blister alveolar cavities. In addition, anemic and normally perfused areas alternate in the lungs while the alveolo-capillary membrane is damaged. The intensity of this injury is proportional to the duration of the drowning process [24].

Other signs of pulmonary hyperinflation are washed-out rhexis bleedings under the pleura, called Paltauf’s spots, named after their first describer [25].

In contrast to freshwater drownings, an osmotic liquid redistribution into the alveolar space with hypertonic hyperhydration occurs during saltwater drowning, resulting in a so-called edema aquosum. This is related to an increase in lung weight [21]. In addition to the changes in the lungs, hypoxia and hypernatremia represent the substantial dangers [2].

Despite the large number of drowning signs listed here, the diagnosis of death by drowning based on autopsy findings is difficult and often remains a diagnosis of exclusion [26]. No finding on its own guarantees a high level of diagnostic accuracy [16, 27]. Typical signs, such as emphysema aquosum or foam in the airways, were not found to be specific for a drowning death in case-control studies. Other autopsy findings only suggest that the body had been exposed to water. In the end, some water corpses do not even show enough morphological findings to determine the exact cause of death [8, 28, 29]. In addition, numerous influencing factors are conceivable. The falsification of the Svechnikov sign in case of putrefaction is only one example [30].

In forensic literature, new methods are described to improve the diagnosis of drowning death. This includes the “aortic hemolytic staining,” a reddish imbibition of the aortic vascular wall, formed by released hemoglobin because of hemolysis [31]. Even so, this method is not specific to drowning alone, as similar observations could be made in cases of putrefaction, sepsis, or burning injuries [31].

Supplementary microscopic examinations are available in the diagnosis of drowning deaths as well. However, the detection of diatoms is currently questioned critically, as diatoms are ubiquitous and have already been detected in corpses with different causes of death [7].

For the diagnosis of “death by drowning,” the forensic physician must assess whether the autopsy findings are consistent with drowning. Often, the obduction of water corpses presents further pathological findings. The reconstruction of the chronological sequence and the mechanisms that led to death are therefore fundamental. Here, it is important for the forensic physician to estimate the specificity of the drowning signs, which other influencing factors exist, and which other individual factors can affect death by drowning.

The aim of this study is to revalue the frequency of the described drowning signs in context of the literature and to identify other possible factors of influence, based on a large investigation sample of deaths in water. At the same time, detailed information on the specificity of drowning signs will be presented and further findings at obduction of deaths in water will be discussed.

## Materials and methods

This retrospective study is based on the obduction material of the Institute of Forensic Medicine of the University Medicine Greifswald (Germany) from 1997 to 2017 (*n* = 4.305). The obduction books of the specified years were systematically examined for deaths related to water in order to include them in the study. In the obduction books, all yearly autopsy cases are listed chronologically. In addition to personal data of the deceased and information on supplementary examinations ordered in the context of the death investigation, it contains a brief description of the circumstances and the final autopsy diagnosis.

The brief descriptions of the circumstances of these deaths were examined according to keywords indicating a corpse found in water. Complementarily, the final autopsy diagnoses were also examined in order to identify further cases of drowning. This resulted in a pre-selection of autopsy protocols that have been examined individually to confirm the selection criterion.

Regardless of the final autopsy diagnosis and postmortem changes, *N* = 331 deaths in water were recorded, corresponding to 7.7% of the institute’s entire autopsies of the named period. In addition to the autopsy protocols, all other documents attached to the protocol were considered. These included, for example, police reports or those of a forensic postmortem examination on site, missing person’s reports, emergency medical records, and patient files of the deceased. Furthermore, the results of all additional forensic examinations were taken into account.

Based on the variables to be examined, a data table was prepared in IBM SPSS Statistics 23 (IBM, Armonk, NY, USA) to capture data of the autopsy protocols. In addition to general information of the autopsied corpses (age, sex, circumstances), it was examined whether the following drowning signs were present: foam at time of corpse recovery, foam at time of autopsy and its location, emphysema aquosum, Svechnikov sign, diluted intestinal contents, Wydler’s sign, gastric mucosal lesions, Paltauf’s spots, hemorrhagic respiratory muscles, blood-stained mastoid processes, and aortic hemolytic staining. Furthermore, the estimated time in water, the time interval until obduction, resuscitation attempts, the progress of putrefaction, and the documented cause of death were recorded.

Most variables could be transferred directly from the autopsy protocol to the dataset. The estimated time in water was only calculated if reliable information was available in police reports or if the sequence of events could be reconstructed. Usually, the specified time a person was considered missing was used as laytime in water. Regarding the circumstances of death, many precise subtopics were first created, and then subsequently summarized into the final groups (see “Results”). Moreover, cases were declared as suicide only if the investigative files provided clear indications (e.g., farewell letter or psychiatric pre-existing condition with suicidal tendency). Specifications on emphysema aquosum were provided only if no other genesis (e.g., chronic emphysema) was mentioned in the protocol. If this was the case, no specification was given here.

If the autopsy showed evidence of longer pre-existing, morbidity-related organ changes (e.g., coronary arteriosclerosis), this was documented as “secondary autopsy diagnosis.” Similar to the approach employed to the circumstances of death, many initially very precise subtopics were summarized to higher level categories such as “cardiac findings.”

If information in the graphs and tables shows a difference to the total number of *N* = 331 cases or to the existing number of cases which met the inclusion criteria in this category, the missing cases could not be specified in the corresponding category.

Within the cohort of all cases of death in water (*N* = 331), further subgroups were formed for a comparative analysis. Based on the final autopsy diagnoses, the cases were classified into the groups *classical drowning*, *atypical drowning*, and *no drowning*. In this study, atypical drowning is defined as a shortened drowning process independent of the shortening reasons. Among them, the frequencies of drowning signs and other autopsy findings were compared. Similarly, for the categories resuscitation (*positive*/*negative*), time in water (*up to 24 h*, *over 1 to 3 days*, *over 3 days to 1 week*, *over 1 week to 1 month*, *over 1 to 3 months*, *over 3 months*) and putrefaction progress (*no*, *beginning*: incipient green discoloration, *proceeded*: extensive discolor and veins visible, *high degree*: putrefaction gas bloated). For the analysis of drowning signs, only corpses after death by drowning (classical or atypical) without putrefaction changes were included (*n* = 199).

The collected data were analyzed using the program IBM SPSS Statistics 23. Descriptive statistics on frequency distributions were compiled. Crosstab tables over relative frequencies were created for the comparative analysis of categorical variables. A significance check was performed with Pearson-*Χ*^2^ test for a fixed significance level of *α* = 0.05. If results are shown as significant in figures, this is always based on a contingency analysis using crosstab tables.

For normally distributed metric variables, such as the amount of liquid content in the sphenoid sinus, the groups’ mean values were compared with a univariate ANOVA and Bonferroni post hoc test. For the category resuscitation, a two-sided *t* test was performed. Again, the fixed level of significance in both was *α* = 0.05.

## Results

### Age, gender, circumstances

The gender ratio was approximately 3:1 in favor of men (75.2% male; 24.8% female). The age at death varied between 1 and 90 years, with an average age of 48.9 years and median of 50 years, while the age was recorded in total years. The average age of women was significantly higher (56.2 years) than that of men (46.5 years) (*p* < 0.001).

The deaths in water could be grouped into nine main groups depending on the circumstances leading to death. The largest group is formed by death circumstances indicating an accident (72.1%) with falls into the water (21.9%), water sports and boating accidents (19.8%), bathing accidents (13.7%), missing person’s cases (10.3%), accidents at work (3.7%), and vehicle accidents (2.7%). Suicides were also common at 18.2%. Deaths in domesticity (7.3%) and homicides (2.4%) have played a minor role.

### Autopsy diagnoses

In *n* = 311 cases, a final autopsy diagnosis was given in the autopsy protocol. It was not possible to detect a clear cause of death in the 20 remaining cases, mostly due to advanced stages of putrefaction (95.0%). In the *n* = 311 cases with available data, classical drowning was found in 73.3%, atypical drowning in 19.6%, and no-drowning as the cause of death in 7.1% of the cases.

During the obduction, many cases showed indications of pre-existing organ pathologies, which were not directly fatal and are subsequently described as “secondary autopsy diagnoses.” Comparing the relative frequencies of secondary autopsy diagnoses within the groups “classical drowning” and “atypical drowning,” victims who underwent an atypical drowning process showed more often a secondary diagnosis with pathological value (see Table 1). Striking is the accumulation of findings at the cardiovascular system such as “coronary arteriosclerosis” (25.5% vs. 16.1%), “heart hypertrophy” (23.4% vs. 12.2%), and “myocardial infarction scar” (6.4% vs. 2.0%).

**Table 1:** Frequencies of secondary section diagnoses in comparison of classical and atypical drowning

			Type of drowning		Total
			Classical	Atypical	
Secondary section diagnosis	non	Count	139	19	158
		%	67.8%	40.4%	62.7%
	Coronary arteriosclerosis	Count	33	12	45
		%	16.1%	25.5%	17.9%
	Heart hypertrophy	Count	25	11	36
		%	12.2%	23.4%	14.3%
	Myocardial scars	Count	4	3	7
		%	2.0%	6.4%	2.8%
	Vascular malformation	Count	4	2	6
		%	2.0%	4.3%	2.4%
Total		Count	205	47	252
		**%**	100.0%	100.0%	100.0%

Some secondary section diagnoses, especially of cardiovascular origin, showed a higher relative frequency in cases of atypical drowning than in the comparison group of classical drownings. The percent values refer to the total number of cases within the group. Other secondary diagnoses are not illustrated, due to their small case numbers

Cases which were summarized as “cardiac findings” were significantly more common in cases of atypical drowning with a relative proportion of 47.5% than in classical drowning (30.7%, *p* = 0.011). No relevant differences could be found for all other secondary autopsy diagnoses. Therefore, a further representation is omitted at this point.

### External signs of drowning: foam

Foam in the airways occurred frequently at 73.3% of all drowning cases examined at the time of obduction. In *n* = 198 cases recorded, 4.0% showed a fungiform formation of external foam. Assuming a retrograde foam spread from the bronchi to the outer respiratory tract, only residues of foam in the bronchi were found in 3.0% of cases, foam reaching into the trachea in 24.4% and to the larynx in 19.2%, and residues up to the mouth and nostrils were observed in 22.7%.

A fungiform formation of external foam directly after corpse recovery was described in 35.6% of cases, with the premise that information on the detection situation was available.

**Fig. 1:**
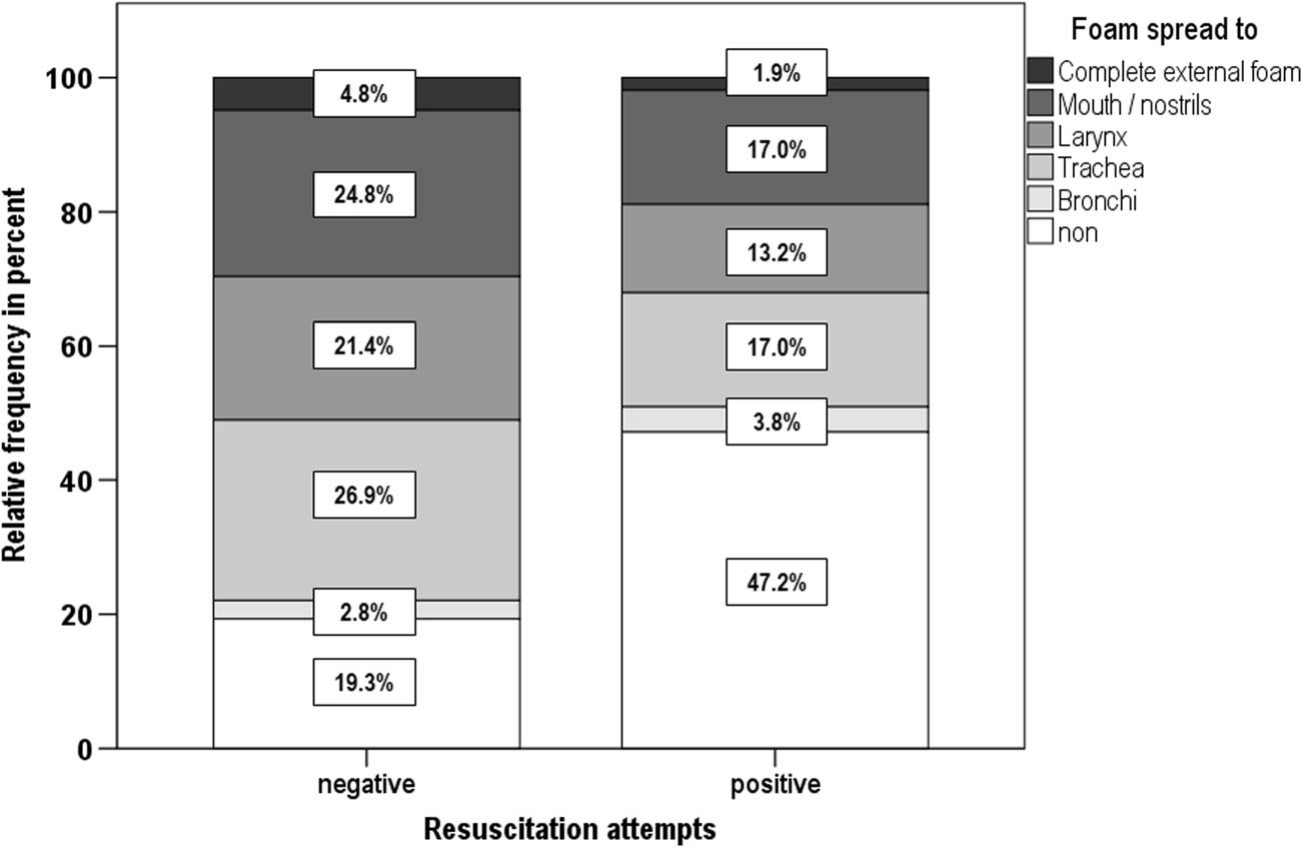
In all other parts of the airways, except the bronchi, the frequency of foam on site, which was spread out less, decreased. The difference is significant at *p* = 0.006

After resuscitation (positive resuscitation = *pR*), no foam was seen more frequent (41.2%) than without resuscitation (negative resuscitation = *nR*, 19.3%). Simultaneously, the appearance of a complete, external foam formation at the time of obduction decreased from 4.8% (nR) to 1.9% (pR). Also, in all other parts of the airways, except the bronchi, the frequency of foam on site, which was spread out less, decreased (see Fig. 1). The difference is significant (*p* = 0.006).

Drowning cases with signs of putrefaction were also included in the analysis of the effect of laytime in water and the incidence of foam. A fungiform formation of external foam was found immediately after recovery at laytime of less than 24 h in 33.9% of cases, at laytime of more than 1 to 3 days in 25.0%, and at laytime of more than 1 week to a month in a single drowning case.

A decrease in the stage of foam expression at the time of obduction could also be documented for long intervals between corpse recovery and obduction, whereby only drowning cases without putrefaction and without resuscitation attempts were included (see Table 2). The table shows that with increasing time intervals between recovery and obduction, the frequency of a complete external foam formation decreased from 12.5 to 3.8%. At the same time, the number of cases without detectable foam increased. If there was foam present, the appearance of foam residues shifted from the mouth/nostrils toward the larynx and trachea.

**Table 2:** Manifestation of foam depending on time interval until autopsy

			Foam spread to						Total
			non	Bronchi	Trachea	Larynx	Mouth/nostrils	Complete external foam	
Time until autopsy	Same day	Count	1	0	1	0	5	1	8
		%	12.5%	0.0%	12.5%	0.0%	62.5%	12.5%	100.0%
	1 day	Count	7	2	14	6	10	2	41
		%	17.1%	4.9%	34.1%	14.6%	24.4%	4.9%	100.0%
	2 days	Count	9	2	8	10	12	2	43
		%	20.9%	4.7%	18.6%	23.3%	27.9%	4.7%	100.0%
	3 or more days	Count	11	0	16	15	9	2	53
		%	20.8%	0.0%	30.2%	28.3%	17.0%	3.8%	100.0%
Total		Count	28	4	39	31	36	7	145
		%	19.3%	2.8%	26.9%	21.4%	24.8%	4.8%	100.0%

The degree of foam expression is demonstrated depending on the time between corpse recovery and autopsy. As seen in the table, in 12.5% of cases, a complete external foam formation was observed at the corpses which were examined on the same day. This decreased to 3.8%, if 3 or more days passed until autopsy. At the same time interval, there was less foam to be detected. Instead of the mouth and nostrils, residues of foam were observed more frequently in the trachea after a correspondingly long time

**Fig. 2:**
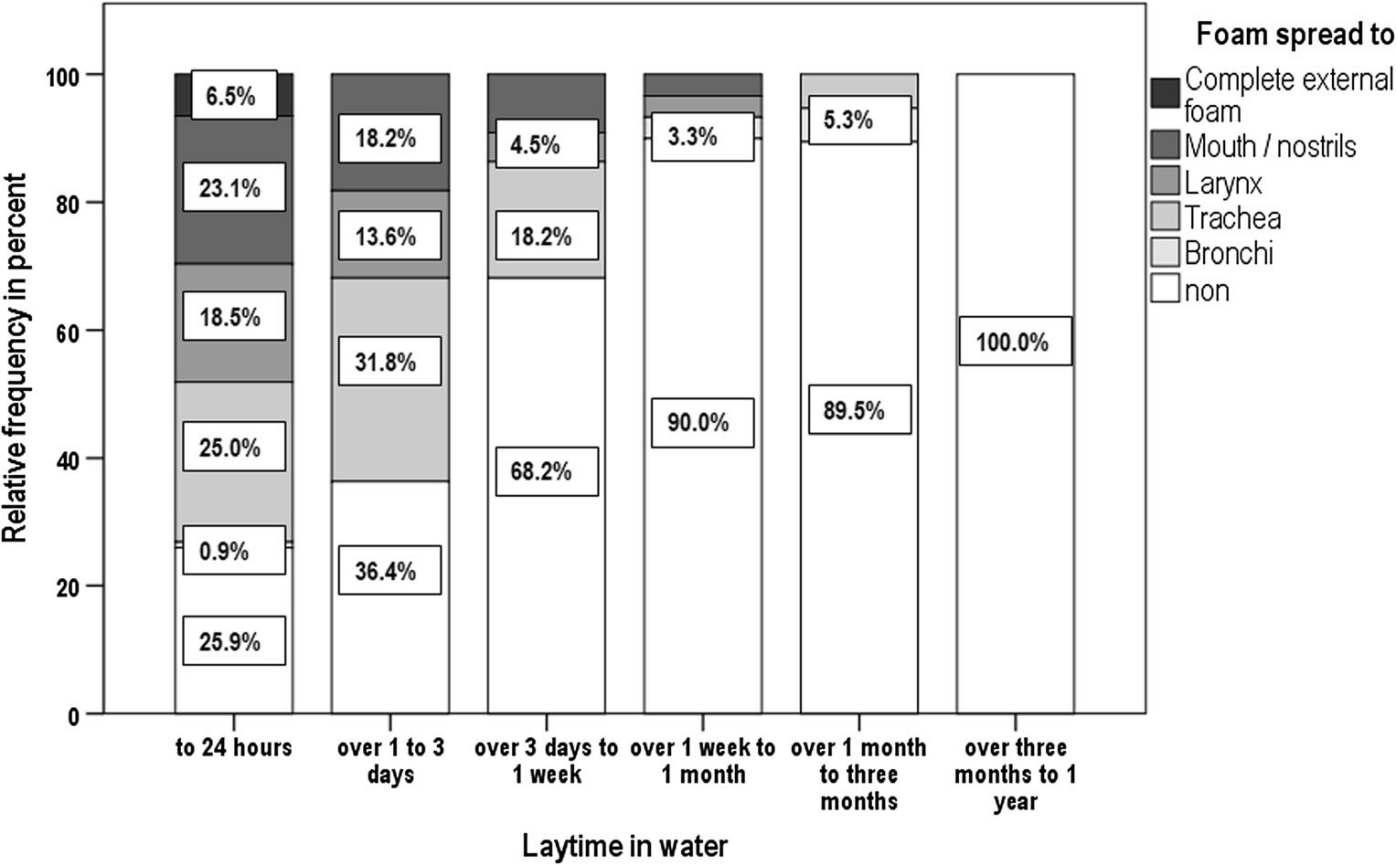
Manifestation of foam depending on laytime in water. The degree of foam expression is illustrated depending on the corpse laytime in water. A complete external foam formation at time of autopsy could only be observed at laytimes of less than 24 h (6.5% of cases). With increasing laytime in water, the frequency of foam detection in autopsy at all decreased. This influence by prolonged laytimes in water is highly significant (*p* < 0.001)

When we analyzed the state of foam expression at the time of obduction in connection to the corpse laytime in water, we also observed a decrease (see Fig. 2). A complete external foam formation was observed only at laytime of less than 24 h (6.5%). With increasing laytime, the frequency of there being any foam decreased significantly (*p* < 0.001).

The effects of putrefaction on the expression of foam were tested for all drowning cases without resuscitation attempts (*n* = 233). At the beginning, putrefaction foam could be detected in 46.7% (vs. 80.7% in case of no putrefaction) of the examined corpses. The more advanced the putrefaction was, the prevalence of foam in the airways continued to decline rapidly from 13.3% (proceeded) to 5.2% (high degree, *p* < 0.001).

**Fig. 3:**
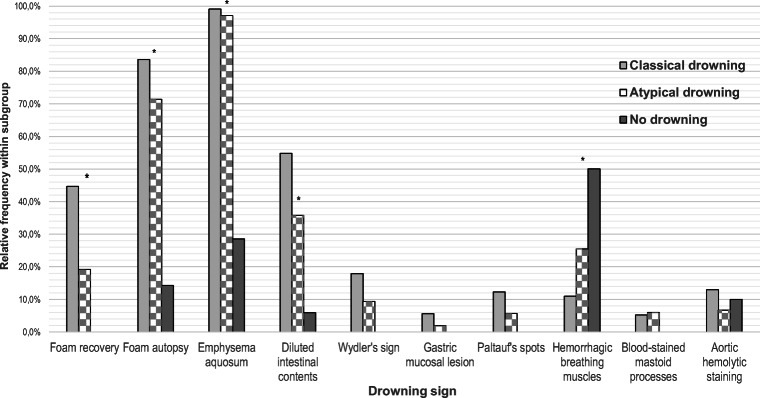
Frequencies of drowning signs within drowning or other causes of death. Of all examined drowning signs, only external foam directly after corpse recovery could be observed significantly and exclusively in cases of drowning. Foam in the airways at time of autopsy was also seen in non-drownings. Other signs, seen exclusively in cases of drowning, occurred in a small number of cases. Emphysema aquosum and hemorrhagic breathing muscles were also common in non-drownings. *Significant distribution

Among the cases without putrefaction (*n* = 218), only cases with cause of death by drowning showed a foam formation after recovery, specifically 44.7% of the classical drowning cases and 19.2% of atypical drowning (*p* = 0.017). In the cases of non-drowning, foam was never documented after recovery (see Fig. 3). On the other hand, foam in the airways at the time of obduction was also observed in 14.3% of the non-drowned victims (83.6% of classical drownings and 71.4% of atypical drownings; see Fig. 3).

### Inner signs of drowning

Emphysema aquosum (94.9%), Svechnikov sign (86.3%), and dilution of intestinal contents (49.7%) have frequently been described in the obduction material. For the Svechnikov sign, whose frequency distribution was determined for all drowning cases with laytime of less than 5 days without putrefaction and without skull injuries (*n* = 117), no positive detection was made in 13.7%, in 12.0% a moisty mucosa, in 21.4% contents up to 1 ml, in 31.6% to 2 ml, in 12.0% to 3 ml, in 5.1% to 5 ml, and in 4.3% over 5 ml of liquid content in the sphenoid sinus.

On the other hand, Wydler’s sign (15.6%), Paltauf’s spots (10.6%), respiratory muscle hemorrhages (14.6%), blood-stained mastoid processes (5.4%), longitudinal gastric mucosa lesions (5.4%), and aortic intimal staining (11.5%) were less common, whereby imbibition of the aortic vascular wall was generally not described in detail (*n* = 122).

Significant influence by resuscitation attempts was shown for the emphysema aquosum (98.6% nR vs. 84.6% pR, *p* < 0.001). In six cases, a return of spontaneous circulation (ROSC) was initially observed. In the later autopsy, edematous lungs were detected.

Wydler’s sign (17.9% nR vs. 9.4% pR, *p* = 0.105), diluted intestinal content (53.4% nR vs. 39.6% pR, *p* = 0.059), Svechnikov sign (mean 1.59 ml nR vs. 1.48 ml pR, *p* = 0.650), respiratory muscle hemorrhages (16.0% nR vs. 11.5% pR, *p* = 0.300), blood-stained mastoid processes (4.4% nR vs. 8.3% pR, *p* = 0.242), and Fritz’ gastric mucosa lesion (4.2% nR vs. 5.8% pR, *p* = 0.447) showed no significant interaction with resuscitation.

Analysis on the influence of the corpse laytime in water, for which cases with putrefaction (*n* = 289) were again included, showed an influence on the emphysema aquosum. For a laytime of less than 24 h, it was found in 99.1%, for more than 3 days to 1 week in 81.8%, for over 1 to 3 months in 70.0%, and for laytime of more than 3 months in 40.0%. For all other signs, no clear negative influence could be identified.

Furthermore, the frequency of emphysema aquosum was negatively affected by putrefaction (analyzed for all drowning cases without resuscitation, *n* = 235). While without putrefaction emphysema aquosum was detected in 98.6% and in 100% of cases with beginning putrefaction, the proportion here decreased to 80.0% with proceeded putrefaction and to 69.5% with high-degree putrefaction (*p* < 0.001).

For the Svechnikov sign, it was found that cases with a high degree of putrefaction showed, with an average of 2.58 ml, a significantly higher amount of liquid content in the sphenoid sinus than cases without putrefaction (mean 1.52 ml; *p* < 0.01). Also negatively affected were the frequencies of respiratory muscle hemorrhages, which could not be detected in drowning cases with advanced and high levels of putrefaction. In cases without putrefaction and with beginning putrefaction, their prevalences were 14.8% and 13.3% respectively (*p* = 0.02). All other findings showed a steady appearance.

Analyses of all corpses without putrefaction (*n* = 218) showed no inner drowning sign, occurring significantly and exclusively in drowning cases (see Fig. 3). A significant increasing number in drowning cases was found for the diluted intestinal contents (54.8% classical drowning, 35.9% atypical drowning, 5.9% no drowning; *p* < 0.001). Other findings, exclusively observed in drowning cases but without statistical significant distribution, were Wydler’s sign (17.9% classical and 9.4% atypical drowning), Paltauf’s spots (12.3% classical and 5.7% atypical drowning), blood-stained mastoid processes (5.2% classical and 6.0% atypical drowning), and gastric mucosa lesions (5.6% classical and 1.9% atypical drowning). On the other hand, the other signs were also very common in the non-drowning deaths (e.g., emphysema aquosum) in water. Some of them (e.g., hemorrhagic breathing muscles) were even more frequently represented in the non-drowning cases (see Fig. 3).

The amount of liquid content in the sphenoid sinus (Svechnikov sign) was significantly higher in cases of classical drowning with an average of 1.64 ml compared to 0.46 ml (no drowning, *p* = 0.046). The mean value for atypical drowning was 1.3 ml.

In our cohort, we found six diving-related cases (three atypical drowning, two typical drowning, one non-drowning [suffocation]). The observed drowning signs in diving-related cases showed no differences to drowning cases of other reasons and no other phenomena.

## Discussion

### Secondary autopsy diagnoses

Morbidity-related pre-existing pathological organ changes of the heart have been found frequently (30.7% classical and 47.5% atypical drowning). Similar results were also provided by studies from Finland (most common secondary findings cardiovascular), Sweden (14% with cardiac damage), and Greece (49% with cardiovascular disease) [32–34]. These findings were significantly more common in the atypical drowning group than in classical drowning cases, leading to the assumption that cardiovascular pre-existing conditions may accelerate the drowning process resulting in less expressed drowning signs. Physiological changes in the circulation, already occurring during immersion, such as a rise in heart rate and increased cardiac output, could lead to decompensation more quickly if cardiac damage is pre-existing [34, 35].

Comorbidities, supporting a death from drowning, are probably a common factor. Cobbett et al. reported concomitant diagnoses which probably led to the death of the person for 38.2% of their autopsy cases of drowning. More than a half of these were cardiac pathologies [36]. Mahony et al. also described 51.6% of all found organ pathologies (predominantly cardiac) as contributory for drowning [37]. Plenzig et al. identified cardiovascular disease as the most important factor in bathtub deaths of victims with pre-existing conditions [35]. These pathologies are assumedly more common in older victims [38]. Mahony et al. postulate that 69% of drowning victims over the age of 65 years have a relevant pre-existing condition [37].

In summary, morbidity, especially in cases of drowning of senior people, should not be underestimated. On the one hand, pre-existing conditions can shorten the time to death onset, and on the other hand, they can also be regarded as a competing cause of death, leading to agonal drowning. The correct interpretation of these secondary findings is therefore challenging.

### External signs of drowning: foam

Frothy liquid and foam in the airways were very common in our investigation of drowning cases (73.3%), but only in 4.0% as a complete, fungiform formation of external foam. In forensic literature, the frequency of external foam is given with 13.5 to 40% [39]. Breitmeier et al. for Hanover and Lunetta et al. for Finland reported frequencies of 17.3% and 14.4% respectively [28, 29]. If data on residues on mouth and nostrils is included, our results are also in the value range as stated above. There are significant differences to an Indian analysis, showing a frequency of 85.8% [40].

A negative correlation between resuscitation attempts and the extent of foam expression was clearly apparent in our study. After resuscitation, no foam detection was significantly more frequent (41.2%) than without resuscitation (19.3%). The frequency of a complete external foam formation decreased from 4.8% (nR) to 1.9% (pR). In a retrospective analysis from Denmark, a complete external foam formation could only be observed if no resuscitation was attempted [41]. Various mechanisms are conceivable to explain this phenomenon. On the one hand, the foam may have been removed by suction [42]. Moreover, displacement mechanisms are also possible due to chest compression, intubation, and ventilation.

This sign was also negatively affected in our analysis by extended laytimes in water and extended time intervals until autopsy. The longer a corpse laid in water, the less foam was still detectable in the airways. We were only able to find a complete external foam formation when laytime was less than 24 h (6.5% of cases). This is consistent with findings by Reijnen et al., demonstrating the highest prevalence for external foam in the time interval between 6 and 24 h post mortem (33% of cases) in their analysis in Amsterdam [43]. Instability of surfactant is claimed to be responsible for the fact that no external foam can be detected after more than 24 h [43]. The lability of the foam also explains the observation that with a prolonged interval between recovery and autopsy, an external foam formation—most often seen immediately after recovery (35.6% of cases with details) or in autopsy at the same day (12.5%)—was seen less frequently.

It can be concluded from our investigations that a fungiform formation of external foam, seen immediately after recovery, may have a high specificity for drowning. This phenomenon could only be observed in cases of drowning. However, this requires that this foam is correctly identified as a drowning sign. Similar foam developments have already been described in other causes of death such as pulmonary edema, drug intoxication, or heart failure and should not be mixed up [13, 44]. However, such specificity does not apply to frothy fluid in the respiratory tract at time of obduction. In our study, foam in the airways was also detected in 14.3% of non-drownings. Reijnen et al. propagate that foam in the airways could be produced by submersion in the water alone, without the need of water aspiration [45]. In a pilot study with deceased piglets, they were able to detect endoscopic foam in the airways after submersion in salt water and fresh water in 35% and 40% of cases, respectively. A fungiform external foam formation was never detected [45].

### Inner sign of drowning: emphysema aquosum

The emphysema aquosum, in German-language forensic literature repeatedly highlighted as an important sign of drowning, has been observed very frequently in our retrospective analysis (99.1% classical drowning; 97.1% atypical drowning). Furthermore, 28.6% of the non-drowned cases showed emphysematous lungs. Breitmeier et al. also reported a frequency of 86.4% for Hanover [29]. Differences were found in studies from South Africa and New Zealand (frequency of 33% and 30% respectively), likely due to the higher importance of drowning cases in saltwater there, causing more often the image of edematous lungs [3, 36].

At the same time, no drowning sign was as much influenced by external factors than emphysema aquosum, whose appearance was reduced by resuscitation attempts, extended laytimes in water, and putrefaction. Due to ventilation under resuscitation, apart from a reduction of this lung sign, as shown in our analysis, theoretically the generation of hyperinflated lungs is also conceivable. A long postmortem interval until the beginning of autopsy is likely followed by an increasing collapse of the lungs. In addition, the dry aspect of the lung cut surface can be concealed by putrefaction fluid.

The correct interpretation of lung changes as emphysema aquosum is important. Experience from our autopsy material shows that hyperinflated lungs often contain additional edematous fluid, which can also be observed in cases of left heart failure, intoxication, or prolonged resuscitation [26]. Furthermore, many deceased people of senior age show emphysematous lung changes regularly, which must be distinguished from emphysema aquosum. Often, only an elaborate histological processing makes a clear distinction possible [46]. Under certain circumstances, lung changes may therefore have been misinterpreted as emphysema aquosum in this study.

Moreover, it is not fully clear to what extent such lung changes can be caused by submersion of a corpse in water alone. In our study, hyperinflated lungs were also found in 28.6% of non-drownings. This is consistent with investigations by Reh et al., who was able to generate the morphological image of “drowned lungs” in not-drowned corpses that had been placed in water [47].

Eventually, the significance and specificity of the emphysema aquosum for the diagnosis of drowning must be questioned. The combination of this lung sign with foam in the airways probably creates higher diagnostic accuracy [48].

### Inner sign of drowning: Svechnikov sign

The Svechnikov sign could frequently be observed (86.3% of drowning cases) in our study material (average of 1.64 ml at classical drownings). This is consistent with results of Bohnert et al., describing Svechnikov signs in 92% of cases, with an average amount of 1.6 ml fluid content [11]. Zivkovic et al. aspirated an average of 1.36 ml liquid out of the sphenoid sinus [30]. Differing data was provided by Hottmar, only describing liquid in sphenoid sinus in 45.7% of cases, and Breitmeier et al., reporting 37.6% [12, 29].

Our research suggests that the Svechnikov sign is not affected by resuscitation attempts or long laytimes in water. However, there seems to be a significant influence due to putrefaction, as with high levels of putrefaction significantly larger amounts of liquid were found in the sphenoid sinus. It is conceivable that this could have been decomposition fluid. Our results contradict the observations of Bohnert et al. and Zivkovic et al., who did not see such a clear influence by putrefaction in their investigations. In the latter study, even less fluid was found in the sphenoid sinuses of decomposed bodies [11, 30].

It should be noted that in drowning cases there were significantly higher amounts of fluid in the sphenoid sinus than in non-drownings (1.65 ml at classical drowning vs. 0.6 ml at non-drowning). For diagnostics, it can be deduced that a quantitative assessment of the Svechnikov sign should also be made [11]. According to our results, this would increase the diagnostic value of the sign.

### Inner signs of drowning: gastrointestinal tract

Findings on the gastrointestinal tract can also be groundbreaking for the diagnosis of drowning deaths. A dilution of the intestinal contents was more common in our study material (49.7%) than Wydler’s sign (15.6%) or gastric mucosa lesions (5.4%). Gotsmy et al. identified a Wydler’s sign in 13% of their autopsy material [19]. Blanco Pampín et al. found 21.1% mucosa lesions in the stomach, essentially as longitudinal tears localized in the corpus or fundus [16].

All gastrointestinal findings appear to be resistant to resuscitation attempts, prolonged laytimes in water, and putrefaction, as these findings could still be described constant. Since autolysis in the stomach starts very early however, it is also conceivable that several lesions could not be seen at time of the obduction [16]. On the other hand, other mucosa lesions may also have been caused by resuscitation attempts, i.e., gastric overinflation by air in cases of false intubation [49].

The specificity of these findings can only be assessed to a limited extent. Although Wydler’s sign and gastric mucosa lesions were observed exclusively in drowning cases, the low frequency of their occurrence precludes reliable information. A significant distribution with increasing numbers in drowning cases was found for the diluted intestinal contents, but 5.9% of the non-drownings showed diluted intestinal content as well. In summary, there is no diagnostic certainty for the gastrointestinal findings, but they could be an important supplement at long postmortem intervals.

### Inner drowning signs: other

For all other drowning signs we examined, the number of cases was too low to provide reliable information on possible influencing factors. According to forensic literature, Paltauf’s spots should be observed in 5–60% of cases, which is consistent with our results (10.6%) [39]. Paltauf’s spots are characterized by their blurred aspect and must be distinguished from other subpleural bleedings, such as Tardieu’s spots.

Hemorrhages into the respiratory muscles were not only rare but could also be observed at non-drowned persons. At the same time, our study showed limited accessibility in cases of advanced putrefaction. Püschel et al. found similar hemorrhages in 34.4% of their examined drowning cases [50]. In case of discovery, such bleedings may be included in the considerations for determining the cause of death [50]. However, they are more unusable for hardening the cause of death by drowning due to their many interpretations (e.g., produced by hypostase) [51, 52].

The aortic intimal staining had not been used yet during our investigation period. The information given here is based only on routine descriptions of the aortic vessel. Reddish imbibition of the vascular wall can for example also be observed in stages of advanced putrefaction [53].

## Conclusion

In summary, it can be concluded from this work that morbidity is a major precursor for death by drowning. Pathologies of the heart can support drowning death and shorten the drowning process in case of incidents in water. The boundaries between natural death in water and an atypical drowning process are fluid. Under certain circumstances, only the obduction can detect such pre-existing conditions and organ pathologies.

Regarding the assessment of our findings, it should be noted that these were not collected directly on the corpse during autopsy but are based on information from the autopsy protocols. Notably, the exact time in water can only be estimated. Individual divergences in the interpretation and description of findings by different coroners could not be avoided.

Furthermore, due to the local occurrences, all deaths contained in the study material took place in freshwater and brackish water, so that the effects of salt water could not be assessed.

Nevertheless, it can be stated that none of the drowning signs we examined guarantees high diagnostic certainty. However, the simultaneous occurrence of multiple drowning signs and the precise exploitation of their benefits can be helpful [28, 29]. For example, at a long postmortem interval, the selective use of findings with high stability is indicated. Findings with high specificity (e.g., external foam formation) increase reliability as well. For the Svechnikov sign, the amount of liquid content should also be quantified and evaluated in this context.

The interpretation must always be carried out to the exclusion of differential diagnoses (quality of foam, morphology of subpleural hemorrhages) and considering the influencing factors described herein. In order to better identify falsification factor due to resuscitation attempts, it would be helpful to leave appropriate equipment in the corpse after unsuccessful resuscitation [49]. A rapid start of autopsy after recovery of the corpse avoids an unnecessary extension of the postmortem interval.

## Data Availability

The datasets generated during and/or analyzed during the current study are available from the corresponding author on reasonable request.
